# In-hospital and web-based intervention to counteract vaccine hesitancy in very preterm infants’ families: a NICU experience

**DOI:** 10.1186/s13052-021-01129-x

**Published:** 2021-09-16

**Authors:** Antonio Di Mauro, Federica Di Mauro, Chiara Greco, Orazio Valerio Giannico, Francesca Maria Grosso, Maria Elisabetta Baldassarre, Manuela Capozza, Federico Schettini, Pasquale Stefanizzi, Nicola Laforgia

**Affiliations:** 1 Pediatric Primary Care, National Pediatric Health Care System, Via Conversa 12, Margherita di Savoia, BAT Italy; 2 Department of Prevention, Local Health Authority of Bari, Bari, Italy; 3 Neonatology and Neonatal Intensive Care Unit, Department of Biomedical Science and Human Oncology, University of Bari, Bari, Italy; 4 Department of Prevention, Local Health Authority of Taranto, Taranto, Italy; 5 Postgraduate School of Public Health, Department of Biomedical Sciences for Health, University of Milan, Milan, Italy; 6 Department of Biomedical Sciences and Human Oncology, University of Bari, Bari, Italy

**Keywords:** Preterm newborn, Vaccine hesitancy, Web-based intervention, Vaccination

## Abstract

**Background:**

Vaccine hesitancy is a global problem, carrying significant health risks for extremely vulnerable population as that of preterm infants. Social media are emerging as significant tools for public health promotion. Our aim was to evaluate both the coverage and the timeliness of routine immunizations in a cohort of preterm infants (**<** 33 weeks of gestational age) at 24 months of age whose families have been subjected to in-hospital and web-based interventions to counteract vaccine hesitancy.

**Methods:**

For a period of 2 years parents of preterm infants were instructed during their follow up visits by a member of the NICU team to get correct informations about vaccines from a social network page. Vaccination rates of preterm infants were assessed at 24 months of chronological age with an electronic database and compared to both general population and historical cohort.

**Results:**

Coverage and timeliness of vaccinations at 24 months of age of 170 preterm infants were analyzed in December 2019. Gestational age and birth weight median (IQR) were, respectively, 31.0 (5.0) weeks and 1475.0 (843.8) g. Coverage rates were similar to those of the regional population (*p* > 0.05), while timeliness of administration was significantly delayed compared to the recommended schedule (*p* < 0.001). Age of administration was not correlated with either body weight and gestational age at birth (Spearman rank, *p* > 0.05). DTaP-IPV-HBV-Hib 2nd and 3rd doses, MMR and Varicella vaccines coverage data were higher compared to historical cohort (*p* < 0.05).

**Conclusion:**

Increasing vaccine confidence through web-based interventions could have a positive impact on vaccination acceptance of parents of preterm infants, although timeliness results still delayed. There is a strong need to develop different and effective vaccination strategies to protect this very vulnerable population.

## Background

Vaccines represent an effective and money-saving health intervention to prevent childhood morbidity and mortality from communicable diseases. Preterm infants vaccinations are particularly important due to their increased susceptibility to infections. Recent data have shown that in preterm newborns even if the vaccine schedule has the same timeliness of term infants [1], vaccinations are often delayed [2]. Despite indisputable vaccines efficacy, fake news and misinformation through mass media and social networks, mainly during last decades, have produced vaccine hesitancy of parents with refusal or delay of vaccinations, causing outbreaks of vaccine-preventable illnesses [3–6].

In 2019 vaccine hesitancy have been identified as one of the top ten threats to global health by the World Health Organization (WHO) and the need to develop effective interventions was pointed up [7].

In this scenario, the role of social media in spreading true scientific informations has earned a growing interest. Despite some authors believe that social media facilitate the spread of fake news, a key barrier to vaccination [8, 9] others suggest that they can provide low-cost, easily and broadly accessible ways to increase vaccine acceptance and to deliver public health messages [10].

Preterm infants are more susceptible to communicable diseases and severe infections [11]. For these reasons, WHO recommends the same immunization schedule for preterm infants of infants born at term, to avoid longer period of vulnerability, in case of timeliness based on corrected age or a supposed target weight.

Despite this recommendation, several studies have shown lower and delayed vaccination rates due to parental vaccine hesitancy in preterm infants [12]. Reasons for under-vaccination of preterm infants are the fear of adverse outcomes or illness caused by the vaccine and lack of awareness of the need for timely vaccinations for this vulnerable population [13]. Moreover, insufficient knowledge of indications for preterm newborns by pediatricians (i.e. the “dilemma” of considering corrected or chronological age or the wrong idea of a target weight to start vaccinations), might have had significant negative effects on the time course of routine immunizations. Vaccination rates have been found lower and delayed in infants of lower gestational age and birth weight [2].

Since 2016, the Neonatal Intensive Care Section of the University of Bari, Italy, has implemented and managed a web-based strategy to increase vaccine confidence for parents of preterm infants through a *Facebook page* named *“UOC di Neonatologia e Terapia Intensiva Neonatale del Policlinico di Bari”* . The Facebook page posted on a regular basis vaccine-related scientific data to convince hesitant parents to do vaccinations on time. NICU team elaborated informations on vaccine with an interactive approach to get active participants and non-stop dialogue.

Facebook posts were arranged into short, easy-to-read paragraphs, discussing risks and benefits of vaccines, information on vaccine-preventable diseases and vaccine safety.

Several other targeted interventions have been also implemented in our NICU to counteract vaccine hesitancy of preterm infants, as already described in a previous work [2]. All stable preterm infants, still hospitalized at chronologic age > 60 days, receive in-hospital vaccinations; parents of home-discharged preterm infants were informed to follow the same routine immunization schedule of term newborns, i.e. chronological and not corrected age; moreover, the outpatient follow-up service provides vaccination counselling.

The primary aim of this study is to evaluate coverage and timeliness of routine immunizations and possible correlations with weight and gestational age at birth in a cohort of preterm infants born from 2016 to 2017, regularly followed in our out-patient clinic up to 2 years of life.

The secondary aim is to evaluate the effect of online dissemination of vaccines scientific data through social media on parental vaccine confidence comparing immunization rates of the interventional 2016–2017 cohort with an historical preterm cohort (2013–2014) not exposed to web-based interventions [2] and the regional pediatric population cohort (2016–2017).

## Methods

### Population included and setting

We performed a prospective study to evaluate, at 2 years of age, coverage and timeliness of routine immunizations of a cohort of preterm infants born in “Policlinico di Bari” from January 2016 to December 2017, and cared first in NICU and then at our out-patient clinic.

Inclusion criteria were: birth before 33 weeks of gestational age, parental internet access and Facebook account.

One hundred ninety-four preterm infants have been admitted in our NICU during the study period. 10 (5,2%) died in their first 2 years of life and 14 (7,2%) lost to follow-up were excluded. 170 (87,6%) met all inclusion criteria: M: 84 (49%), F: 86 (51%) with median (IQR) gestational age and birth weight, of 31.0 (5.0) weeks and 1475.0 (843.8) g, respectively.

### Intervention

According to the Italian vaccination schedule all infants before the 24th month of life receive the following vaccines against: Hexavalent (Diptheria, Pertussis, Tetanus, Poliovirus, Hepatitis B and *H. influenzae* type B); Meningococcus C and B; Pneumococcus; Rotavirus, Tetravalent (Measles, Mumps, Rubella and Varicella). In our region, recommended vaccines for routine immunizations within 24 months of life includes 3 doses of Hexavalent vaccines at 61, 151 and 331 days of life, one dose of Tetravalent at 12 months of life, three doses of conjugate pneumococcal vaccine at 61, 151 and 331 days of life and one dose of conjugate meningococcal C at 12 months of life. All these vaccines are free of charge for the entire population.

All stable preterm newborns still admitted at the age of 61 days of life, were vaccinated with hexavalent, Meningococcus C and Pneumococcus before discharge, while, our medical personnel give indications to all parents of the immunization program, the same of full-term infants, regardless of birth weight and gestational age at birth, at preterm discharge before 61 days of life.

Vaccination counselling was scheduled during all outpatient visits at 2, 4, 6 and 12 months of age and the medical personnel give instructions to parents to follow the NICU Facebook page and interact with the posts and all the activities to increase their knowledge about vaccines. .

On NICU *Facebook* page, the research team posted short and easy-to-read contents, with information about risks and benefits of vaccines, vaccine-preventable diseases and the recommended immunization schedule. Parents were allowed to post comments and questions and to get answers from the team.

Immunization data for all preterm infants of their first 24 months of life, were extracted in December 2019 from the electronic regional vaccination database (GIAVA). Immunization status and timeliness of administration were assessed for the following vaccines: Hexavalent, pneumococcal conjugate; meningococcal C; Tetravalent.

### Statistical methods

Statistical analysis was performed using R version 4.0.0 (released on 2020-04-24). Statistical significance α was fixed to 0.05. Categorical variables (vaccine coverage) were reported as absolute and relative frequencies and compared through z test for proportion. Numerical variables (age at vaccine administration) non normally distributed (Shapiro-Wilk test) were reported as median (IQR), compared through Wilcoxon Rank test and, in order to assess their correlation with weight and gestational age at birth, Spearman rank correlation coefficient Rho was calculated (*P*-values were computed via the asymptotic t approximation).

## Results

Vaccinations coverage and timeliness were assessed at 24 months of age and 2016–17 preterm cohort and 2016–17 regional general population are reported in Table 1.

**Table 1:** 2016–2017 vaccine coverage in preterm infants and regional paediatric population (z-test)

Vaccine	Preterm cohort		Regional pediatric population	p
	n/N	% (95%CI)	%	
DTaP-IPV-HBV-Hib 1 doses	169/170	99.4 (96.3–100.0)	99.0	0.878
DTaP-IPV-HBV-Hib 2 doses	165/168	98.2 (94.5–99.5)	94.8	0.069
DTaP-IPV-HBV-Hib 3 doses	161/167	96.4 (92.0–98.5)	95.2	0.583
PCV 1 doses	167/170	98.2 (94.7–99.5)	98.4	> 0.99
PCV 2 doses	160/168	95.2 (90.5–97.8)	94.1	0.644
PCV 3 doses	152/167	91.0 (85.4–94.7)	93.6	0.228
Men C	137/167	82.0 (75.2–87.4)	83.2	0.765
MMR	158/167	94.6 (89.7–97.3)	94.2	0.951
Varicella	158/167	94.6 (89.7–97.3)	91.7	0.221

99.4% (169/170) of enrolled preterm newborns received the 1st dose of hexavalent vaccine and 98.2% (167/170) the 1st dose of pneumococcal conjugate vaccine. The vaccination rates decreased to 98.2% (165/168) and 96.4% (161/167) for the 2nd and 3rd dose of hexavalent vaccine and to 95.2% (160/168) and 91% (152/167) for the 2nd and 3rd dose of pneumococcal conjugate vaccine, respectively. Coverage for MMR, Men C and varicella vaccines were, respectively, 94.6% (158/167), 82.0% (137/167) and 94.6% (158/167).

Overall, vaccination rates of preterms were not significantly different from those of regional general paediatric population (*p* > 0.05).

Average age of vaccine administration in preterm infants and recommended timeline are reported in Table 2. For all vaccines, the age of vaccine administration in preterm newborns was higher than the recommended timeline (*p* < 0.001).

**Table 2:** Age of vaccine administration in preterm group compared to recommended timelines (Wilcoxon Signed Rank test)

Vaccine	Preterm group	Recommended age (days)	p
	Median (IQR)	value	
DTaP-IPV-HBV-Hib 1 doses	99.0 (45.0)	61.0	**< 0.001**
DTaP-IPV-HBV-Hib 2 doses	184.0 (79.0)	121.0	**< 0.001**
DTaP-IPV-HBV-Hib 3 doses	395.0 (101.0)	336.0	**< 0.001**
PCV 1 doses	104.0 (47.0)	61.0	**< 0.001**
PCV 2 doses	183.5 (73.3)	121.0	**< 0.001**
PCV 3 doses	394.5 (85.5)	336.0	**< 0.001**
Men C	506.0 (116.0)	366.0	**< 0.001**
MMR	454.5 (131.0)	426.0	**< 0.001**
Varicella	454.5 (131.0)	366.0	**< 0.001**

Age of vaccine administration showed no correlations with both body weight and gestational age at birth (*p* > 0.05, Table 3).

**Table 3:** Correlation between age of vaccine administration with body weight and gestational age at birth in preterm infants, birth cohort 2016–2017 (Spearman Rank)

Vaccine	Birth weight		Gestational age at birth	
	Rho	p	Rho	p
DTaP-IPV-HBV-Hib 1 doses	−0.102	0.187	−0.080	0.303
DTaP-IPV-HBV-Hib 2 doses	+ 0.004	0.952	−0.019	0.810
DTaP-IPV-HBV-Hib 3 doses	+ 0.053	0.504	+ 0.033	0.682
PCV 1 doses	−0.104	0.182	−0.096	0.216
PCV 2 doses	−0.011	0.891	−0.040	0.612
PCV 3 doses	−0.001	0.999	+ 0.004	0.955
Men C	+ 0.117	0.172	+ 0.155	0.070
MMR	+ 0.065	0.416	+ 0.006	0.943
Varicella	+ 0.065	0.416	+ 0.006	0.943

Vaccine coverage in 2016–17 preterm cohort and historical preterm cohort are reported in Table 4. DTaP-IPV-HBV-Hib 2 and 3 doses, MMR and Varicella shows higher values in 2016–17 group (*p* < 0.05).

**Table 4:** Vaccine coverage of preterm cohorts 2016–17 and historical 2012–14 preterm cohort (z-test)

Vaccine	Preterm cohort		Preterm historical cohort		p
	n/N	% (95%CI)	n/N	% (95%CI)	
DTaP-IPV-HBV-Hib 1 doses	169/170	99.4 (96.3–100.0)	156/159	98.1 (94.6–99.6)	0.568
DTaP-IPV-HBV-Hib 2 doses	165/168	98.2 (94.5–99.5)	145/159	91.2 (85.7–95.1)	**0.009**
DTaP-IPV-HBV-Hib 3 doses	161/167	96.4 (92.0–98.5)	138/158	87.3 (81.1–92.1)	**0.005**
PCV 1 doses	167/170	98.2 (94.7–99.5)	157/159	98.7 (95.5–99.8)	> 0.99
PCV 2 doses	160/168	95.2 (90.5–97.8)	144/159	90.6 (84.9–94.6)	0.151
PCV 3 doses	152/167	91.0 (85.4–94.7)	136/158	86.1 (79.7–91.1)	0.220
Men C	137/167	82.0 (75.2–87.4)	135/157	86.0 (79.6–91.0)	0.414
MMR	158/167	94.6 (89.7–97.3)	120/157	76.4 (69.0–82.8)	**< 0.001**
Varicella	158/167	94.6 (89.7–97.3)	127/157	80.9 (73.9–86.7)	**< 0.001**

Furthermore, comparison of the average age of vaccine administration between the two preterm cohorts shows only earlier administration of the first dose of Men C in 2016–17 preterm cohort respect to the historical 2012–14 preterm cohort (Table 5). No differences were observed for others vaccines.

**Table 5:** Average age of vaccine administration between preterm cohorts 2016–17 and historical 2012–14 preterm cohort (t-test)

Vaccine	Age at administration in preterm cohort	Age at administration in historical preterm cohort	p
	Median (IQR)	Median (IQR)	
DTaP-IPV-HBV-Hib 1 doses	99.0 (45.0)	96.0 (24.5)	0.632
PCV 1 doses	104.0 (47.0)	96.0 (31.0)	0.330
Men C	506.0 (116.0)	547.0 (179.5)	**0.027**
MMR	454.5 (131.0)	459.0 (133.0)	0.536
Varicella	454.5 (131.0)	452.0 (123.5)	0.953

## Discussion

Vaccine hesitancy is a well-known phenomenon described in parents of children with underlying health problems and insufficient vaccine coverage rates have been reported for both children and adults with chronic diseases [14, 15].

Health care providers should offer an open and steady dialogue with parents using appropriate communication tools in order to maintain high immunization rates, especially in vulnerable population.

In 2018, a consensus of Italian experts from the Italian Society of Hygiene and Preventive Medicine and from the Italian Society of Paediatrics and the Italian Federation of Paediatrics raised concerns about the delay of the national vaccination schedule in preterm infants, because of significant cases of pertussis and other vaccine-preventable diseases in this fragile population [16].

The mean reason to delay vaccination in very preterm and/or low birth weight infants seems to be due to concerns about vaccine safety, despite the frequency of adverse events between preterm and term infants is not different [17].

Except for the first dose of hexavalent and PCV, whose administration could be delayed because of the need of starting immunization when preterm infants are clinically stable [18], there is no other reasons to delay the administration of all following doses.

In Europe over the last years vaccine confidence has significantly dropped and only recently it has been reported to raise [8]. Anti-vaccine movements have contributed to increase vaccine hesitancy with a significant threat for public health. These movements have often used social media to spread their fake messages, devoid of any scientific evidence and the scientific community has expressed their concern for the dissemination of fake news with a negative impact on vaccination coverage [19].

Anyway, social media are not only a bad vehicle of fake news; being low cost and easily accessible they could potentially be used to spread scientific data, as demonstrated during the recent pandemic [20, 21].

Our study aimed to evaluate if correct vaccine-related informations through social media could improve vaccination coverage with correct timeliness in preterm newborns. Our data show that vaccination rates at 24 months of age does not differ from those of the general paediatric population; but, despite the efforts to improve timeliness of immunization schedule, age at administration is still delayed for all vaccines investigated. We did not observe any correlation between gestational age and birth weight with time of vaccine administration, differently from other reports showing that vaccine delay is positively related with both lower birthweight or gestational age [2, 22, 23]. We speculate that the correct information about vaccine in preterm newborns is the key role for these positive and unexpected results.

This observational study suggests that spreading vaccine-related scientific data on social media could positively influence vaccine acceptance by parents of preterm infants, increasing vaccine vaccination rates. This finding is in accordance with the study by Glanz et al. that also showed that social media can positively influence vaccine acceptance better than providing plain information or usual care [10]. Furthermore, web-based intervention might influence also the correct timeliness of vaccine administration, as showed in our cohort for anti-MenC.

This finding could be related to the awareness of parents of preterm newborns of the higher incidence and severity of meningitis in this fragile populations with a greater risk of bad neurodevelopmental outcome [24].

According to our results, we would encourage pediatricians and already pediatric residents during their training to became confident in the use of social networks to provide correct and evidence-based informations to Italian families [25]. Furthermore, we would highlight the very important role of qualified healthcare workers of the NICU team, both neonatologists and nurses, to provide right shared informations to parents of preterm infants to enhance their vaccination adherence. We strongly believe that the relationship between health personnel and families, established in the ward or in UTIN, may effectively counteract parental vaccine hesitancy.

Therefore, it would seem useful to emphasize that the empathy and availability of qualified healthcare workers of the NICU team to provide web-based right shared information has a positive impact on parents and on vaccination adherence of preterm infants.

This study has several strengths: the very low lost to follow-up rate (7,2%) and the accuracy of data because of the computerized regional surveillance system (GIAVA) providing a systematic general paediatric control group. There are also some limitations. First, the data of this observational cohort have been compared to an historical cohort from the same center, with no randomized interventions. Furthermore, any difference in vaccination rates between those two cohorts might have been due to other factors different from the web-based intervention. We are aware that RCT trials are needed to confirm that vaccine informations given through social media could positively influence vaccine acceptance of parents of preterm infants. Second, the socio-economic background of parents was neither recorded at baseline nor assessed and it is very interesting to evaluate any difference among families. Finally, participants had unlimited access to social network, but both the time spent and their participation were not recorded and also the trial was conducted in a single NICU, with a specific protocol for vaccination of preterm infants, as already described [2], although this intervention after 61 days of life for still admitted newborns does not represent a routine standard of care in all Italian NICUs.

To the best of our knowledge, this is the first trial aimed to investigate the role of social media in providing scientific vaccine informations for the families of preterm infants. RCT trials with specific surveys among parents may better clarify the role of web-based interventions..

## Conclusion

A scientific-based use of social media, with two-way interaction with users, should be an important tool in the hands of medical personnel to positively influence parental acceptance of vaccines and counteract vaccine hesitancy.

These strategies are particularly needed for the very vulnerable population of preterms, in which any delay of vaccinations increase the risk of infectious disease-related complications.

## Data Availability

The datasets used and/or analyzed during the current study are available from the corresponding author on reasonable request.

## Abbreviations

DTaP: Anti-diphtheria-tetanus-acellular pertussis vaccine
HBV: Anti-hepatitis B vaccine
Hib: Anti-*H. influenzae* type b vaccine
IPV: Anti-polio vaccine
MenC: Meningococcal C vaccine
MMR: Anti-measles, mumps, rubella vaccine
NICU: Neonatal intensive care unit
PCV: Anti-pneumococcal conjugate vaccine
RCT: Randomized controlled trial
WHO: World Health Organization
